# Contrastive analysis on the safety of brand and generic nebivolol: a real-world pharmacovigilance study based on the FDA adverse event reporting system

**DOI:** 10.3389/fphar.2024.1280201

**Published:** 2024-01-31

**Authors:** Hongli Wang, Guizun Zhong, Huanhuan Ji, Siqi Chen, Qinqin Xie, Zhengze Shen, Yuntao Jia

**Affiliations:** ^1^ Department of Pharmacy, Yongchuan Hospital of Chongqing Medical University, Chongqing, China; ^2^ College of Pharmacy, Chongqing Medical University, Chongqing, China; ^3^ Department of Pharmacy Children’s Hospital of Chongqing Medical University, National Clinical Research Center for Child Health and Disorders, Ministry of Education Key Laboratory of Child Development and Disorders, Chongqing Key Laboratory of Child Rare Diseases in Infection and Immunity, Chongqing, China

**Keywords:** nebivolol, original drug, generic drug, FDA adverse event reporting system, disproportional analysis, adverse reaction

## Abstract

**Background:** The equivalence of generic drugs to their brand-name counterparts is a controversial issue. Current literature indicates disparities between the generic nebivolol (GN) and the brand nebivolol (BN).

**Aim:** The study is designed to investigate the safety difference between GN and BN and provide reference information for clinical practice.

**Methods:** We reviewed adverse event (AE) reports that recorded nebivolol as the primary suspect drug in the FDA Adverse Event Reporting System (FAERS) database from 2004 to 2022, conducted a disproportional analysis to detect signals for the GN and BN respectively, and compared the AE heterogeneity between them using the Breslow-Day test.

**Results:** A total of 2613 AE reports of nebivolol were recorded in the FAERS database from 2004 to 2022, of which 2,200 were classified as BN, 346 as GN, and 67 unclassifiable AE reports were excluded. The signals of 37 AEs distributed in cardiac, gastrointestinal, psychiatric, and nervous systems were detected in disproportional analysis. 33 out of 37 AEs were positive signals, with 21 not previously listed on the drug label, indicating an unrecognized risk with nebivolol. In the heterogeneity analysis of AE signals between GN and BN, the GN generally showed a higher AE signal value than BN, especially 15 AEs distributed in the cardiac, neurological, and psychiatric systems that showed statistically significantly higher risk by taking GN.

**Conclusion:** Our study shows some previously overlooked adverse effects of nebivolol. It suggests that the risk of GN’s adverse effects may be higher than those in BN, which deserves further attention and investigation by healthcare professionals, regulators, and others.

## 1 Introduction

Brand drugs are original drugs that undergo extensive testing and clinical trials before being approved for marketing, in which substantial financial resources and time are invested. Correspondingly, generic drugs refer to the equivalent substitute manufactured based on the original drug formula, which is the same as brands in dosage form, safety, strength, route of administration, quality, performance characteristics, and intended use (Rana and Roy, 2015). Since generic drugs usually have a lower cost of running and market prices than brands, they have accounted for a significant share of the global pharmaceutical market, accounting for 86% in the United States, 68.6% in Canada, and 17%–83% in Europe (Bayram et al., 2021). Generic drugs have indeed become the cornerstone for providing affordable medicines to patients. However, although generic drugs and brands have the same active ingredient, generic drugs may be different from brands in an inert binder, tablet color, and manufacturing process, which may result in variations in safety profiles (van der Meersch et al., 2011; Andrade, 2015). Meanwhile, studies have demonstrated that the generic drugs may not be clinically equivalent to brands. For example, one study showed that generic clopidogrel might have a higher safety risk in real-world than the original drug, and another study showed that the development of seizures or unexpected may occur when brand antiepileptics such as sodium valproate and lamotrigine are switched to the generic drugs (Bialer and Midha, 2010; Serebruany et al., 2019). Therefore, clinicians, scientific societies, and patients have expressed many concerns about generic drugs’ long-term efficacy and safety and the consequences of potentially multiple switches being dictated by economic pressure rather than medical needs (Sarzi-Puttini et al., 2019). Generic drugs typically have shorter development cycles and they are approved for clinical use based on small bioequivalence studies. The inherent limitations of generic drugs’ development make them invariably focus on observing effectiveness indicators and need long-term or large-sample safety studies (Glerum et al., 2020). Therefore, it is necessary to continue to pay attention to the difference in efficacy and safety between generics and brands and explore feasible evaluation strategies.

Nebivolol is a novel beta-blocker (β-blocker) approved by the US Food and Drug Administration (FDA) in 2007, which exhibits highly selective in β1 receptors and exerts unique pharmacological properties by activating the nitric oxide synthase (NOS) pathway by activating β3 receptor in the endothelium (Fongemie and Felix-Getzik, 2015). Compared with other β-blocker, nebivolol has certain advantages in the treatment of hypertension, including the significant improvements in endothelial dysfunction, central hemodynamics, the degree of erectile dysfunction in men, a beneficial metabolic profile, and a more favorable side effect profile (Olawi et al., 2019). In recent years, the generic nebivolol (GN) has been emerging. However, there is still a lack of comparative data between GN and brand nebivolol (BN) to guide clinicians in deciding whether generic substitution is appropriate. Moreover, a study that compared the difference between GN and BN in pharmacokinetic and pharmacodynamic properties attracts our attention. The study showed that although the comparison of the pharmacokinetic parameters of GN and BN met the criteria, a difference existed in the impact on the heart rate of the subjects between them (Bambysheva et al., 2016), which stimulated our interest in further exploring the difference in efficacy and safety between GN and BN.

Pharmacovigilance is the science and activities relating to the detection, assessment, understanding, and prevention of adverse effects or any other possible drug-related problem, and the establishment and application of a pharmacovigilance database is an essential integral part of it (Beninger, 2018). The pharmacovigilance databases are widely used to conduct post-marketing surveillance of drugs in the real world and to provide the public with information on possible adverse drug events (AEs). In this regard, the FDA Adverse Event Reporting System (FAERS) database, a database with a large population, comprehensive geographic coverage, and publicly available accessibility, has become one of the essential data sources that is commonly used for research in the field of pharmacovigilance (Li et al., 2023). Meanwhile, previous literature has also confirmed the feasibility of using FAERS to explore safety differences between generic drugs and brands (Rahman et al., 2017b; Cheng et al., 2018). In this study, we reviewed and analyzed the AE data in the FAERS database to investigate drug safety differences for BN and GN, expecting to provide health professionals and patients with information on drug safety for clinical use and selection.

## 2 Methods

### 2.1 Data source

The FAERS database is generated from the FDA’s post-marketing safety surveillance program. It contains AE and medication error reports submitted by healthcare professionals, consumers, manufacturers, or others aware of AEs in patients. The AE data in the FAERS database is highly structured and available, and all the AEs are converted to standardized terminology called Preferred Term (PT) using the Medical Dictionary for Regulatory Activities (MedDRA). The FAERS database has publicly opened more than 10 million AE reports received by the FDA since 2004 and is updated quarterly. In this study, we used OpenVigil 2.1 (http://h2876314.stratoserver.net:8080/OV2/search/), an open tool for data mining and analysis of pharmacovigilance data using cleaned FAERS adverse event reports, to retrieve and extract the structured data of nebivolol in the FAERS database from the first quarter of 2004 to the fourth quarter of 2022 (Li et al., 2022).

### 2.2 AE reports extraction, processing, and differentiation

The present study investigated the BN and GN in the FAERS database. Firstly, we extracted all the raw data of AE reports containing nebivolol in the FAERS database from 2004 to 2022 through OpenVigil 2.1. Secondly, to accurately collect the AE reports mainly attributed to nebivolol, we screened out the reports with nebivolol as the primary suspect according to the recorded role of nebivolol in the AE report. Thirdly, we went through each AE case according to the safety report ID (ISR) number and reviewed the trade name, manufacturer, and new drug application (NDA) or abbreviated new drug application (ANDA) number to classify nebivolol into BN and GN. If the nebivolol is recorded with the tradename “BYSTOLIC,” the NDA number “021742,” or the submitter is from Allergan or Frost, we consider it the BN. On the contrary, if the submitter is from Ani, Alkem, Watson, Glenmark, Hetero, Indchemie, Torrent, Micro Labs, Cadila, Aurobindo, Prinston, Reyoung, Ajanta, Unichem, Mankind, Beximco, or the ANDA number is the same as the generic drug on file (https://www.accessdata.fda.gov/scripts/cder/daf/index.cfm?event=overview.process&ApplNo=021742), we consider it as the GN. When the AE report is indistinguishable, we exclude it. Finally, according to the ISR number, the report characteristics of BN and GN were counted, including patient age, sex, and AE outcomes.

### 2.3 Data statistics and analysis

To determine the target ADR for analysis, we searched the most common ADRs based on literature and the label from the FDA official website. The most common AEs were headache, dizziness, nausea, diarrhea, tiredness, and bradycardia, of which bradycardia, nausea, and headache may lead to discontinuation (Riva and Lip, 2011; Hanif et al., 2023). We mapped the above ADRs to their primary system organ class (SOC) according to MedDRA 26.0, mainly involving nervous system disorders, gastrointestinal disorders, and cardiac disorders. Moreover, highly lipid-soluble β-blockers are centrally enriched in the central nervous system and may lead to psychiatric disorders (Kumar et al., 2007), and this SOC is also the one that the label of nebivolol focuses attention on. Therefore, this study explored the AE signals in the above four SOCs to compare the safety difference between GN and BN.

In this study, the reporting odds ratio (ROR), a well-established algorithm of disproportional analysis method, was used to detect ADR signals. The two-by-two contingency table used for the calculation is shown in Table 1, and the ROR value and its corresponding 95% confidence interval (CI) can be calculated by following the formula (Sakaeda et al., 2013):

$$\text{ROR}=\frac{a/c}{b/d}=\frac{ad}{bc}$$ (1)

$$95\%\mathrm{CI}=e^{\ln\left(ROR\right)\;\pm 1.96\sqrt{\left(\frac{1}{a}+\frac{1}{b}+\frac{1}{c}+\frac{1}{d}\right)}}$$ (2)

**TABLE 1:** The two-by-two contingency table for disproportional analysis.

	Drug of interest	Other drugs	Total
AEs of interest	a	b	a+b
Other AEs	c	d	c + d
Total	a+c	b + d	a+b + c + d

AEs, adverse events.

A positive ADR signal is defined as the number of AE reports greater than or equal to 3 (a ≥3 in Table 1) and the lower-bound 95% CI of ROR value greater than 1, while a negative signal is defined as the number of AE reports or the lower-bound 95% CI of ROR value cannot reach above criterion (Li et al., 2023). Besides, referring to previous literature (Rahman et al., 2017a), the Breslow-Day test was used to test the heterogeneity of ROR between GN and BN, and significantly statistical difference was existed when *p* < 0.01.

## 3 Results

### 3.1 Descriptive analysis

**FIGURE 1:**
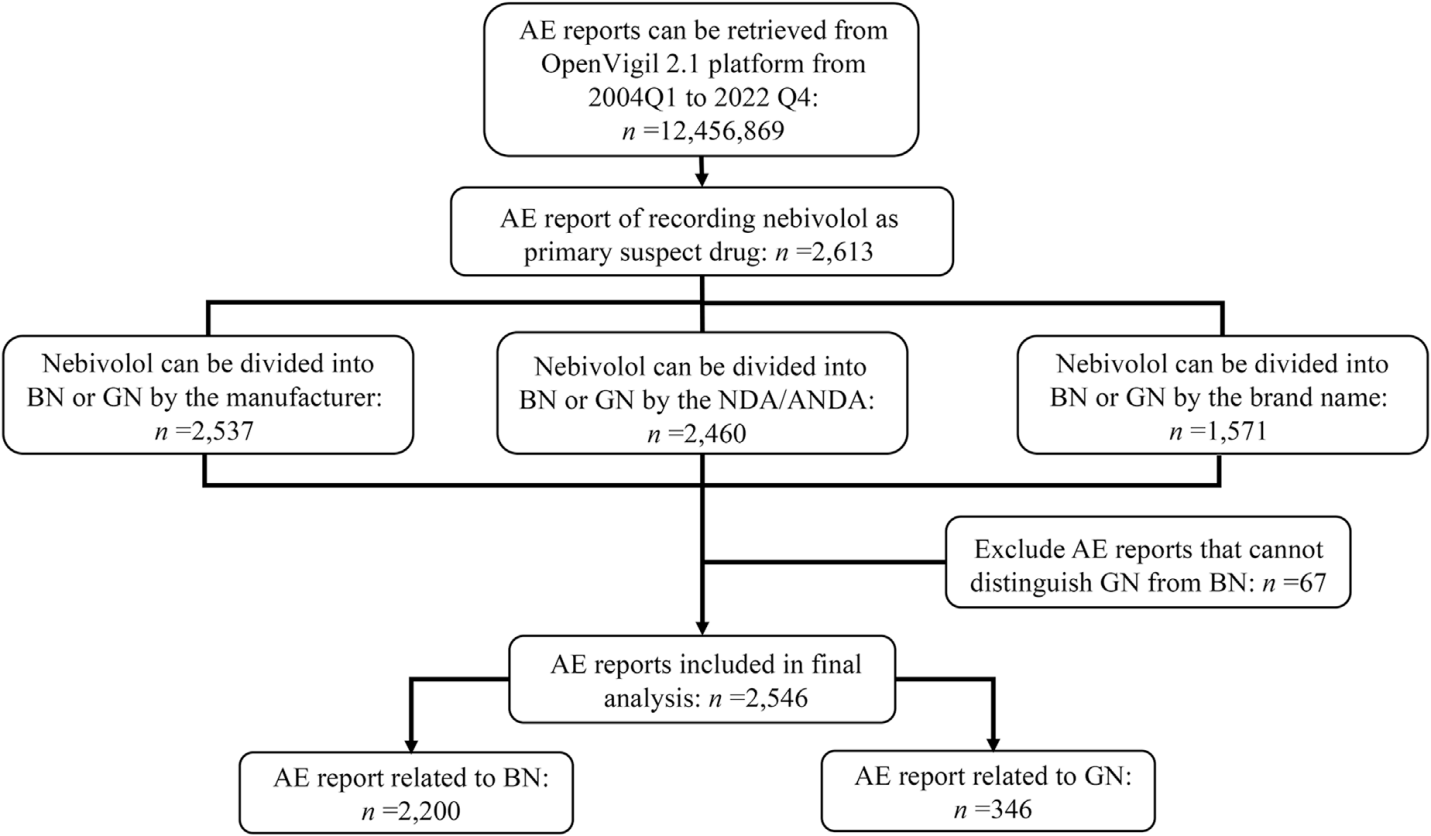
Flowchart of target reports identification and reporting subgroup summarization. Abbreviate: Q, quarter; NDA, new drug application; ANDA, abbreviated new drug application; BN, brand nebivolol; GN, generic nebivolol.

12,456,869 AE reports were retrieved using the OpenVigil 2.1 platform between the first quarter of 2004 and the fourth quarter of 2022, in which 2,613 reports recorded nebivolol as the primary suspect drug. Further, examining the corresponding trade names, NDA/ANDA number, and manufacturer of nebivolol, 67 reports that cannot be distinguished between GN and BN were excluded. Of the remaining 2,546 ADR reports, 2,200 were classified into BN, and 346 were classified into GN. The identification details of GN’s and BN’s reports are shown in Figure 1. The demographic features of those reports are tallied in Table 2. Among patients suffering AEs of nebivolol, BN and GN share the same trend in gender, with more females than males, and the patients are mainly aged (>64 years old). Regarding patient outcome, the outcomes of cases affected by BN and GN were mainly hospitalization-initial or prolonged, accounting for 334 (15.18%) and 161 (46.53%), respectively.

**TABLE 2:** Demographic characteristics of patients for nebivolol.

Parameters	BN		GN	
	Number of AE reports (N = 2200)	Ratio (%)	Number of AE reports (N = 346)	Ratio (%)
Gender				
Female	1186	53.91	135	39.02
Male	766	34.82	133	38.44
Information missing	248	11.27	78	22.54
Age group				
<18	51	2.32	37	10.69
18–44	127	5.77	22	6.36
45–64	443	20.14	68	19.65
>64	564	25.64	132	38.15
Information missing	1015	46.14	87	25.15
Patient outcomes				
Hospitalization or prolonged	334	15.18	161	46.53
Death	68	3.09	16	4.62
Life-Threatening	42	1.91	29	8.38
Disability	17	0.77	3	0.87
Congenital Anomaly	2	0.09	9	2.60
Other serious conditions	546	24.82	181	52.31

A report contains more than one outcome. BN, brand nebivolol; GN, generic nebivolol.

### 3.2 AE signal detection results

**FIGURE 2:**
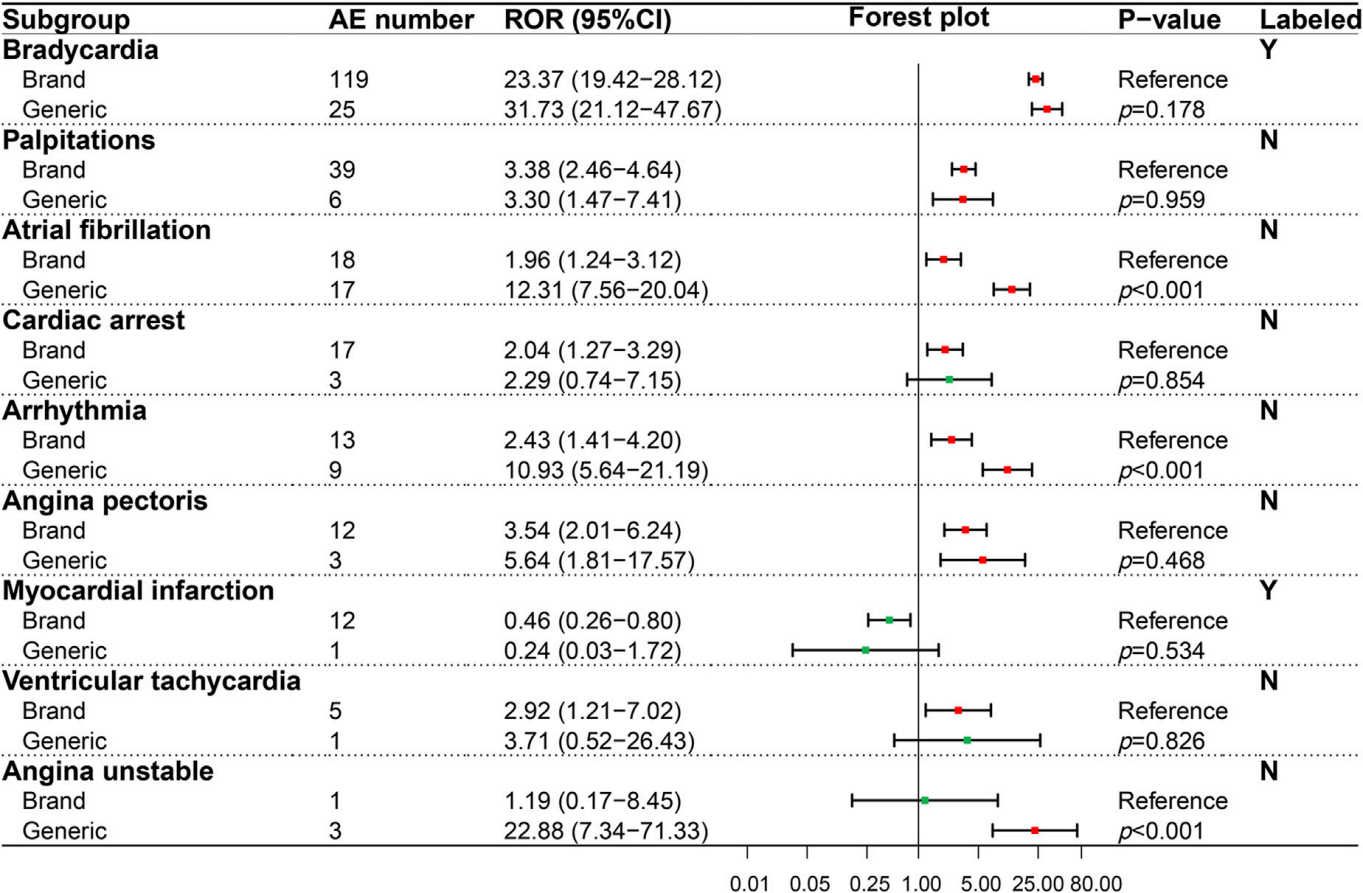
Comparison of detected AE signals for brand and generic nebivolol in the cardiac system. Abbreviate: AE, adverse event; ROR, reporting odd ratio; CI, confidence interval. Note: Red points indicate positive signals and green points are opposite; the *p*-value results from the Breslow-Day test; label information comes from the US Food and Drug Administration (FDA) official website.

In our study, we detected 37 AE signals in four interested SOCs by the ROR method and compared the statistical differences of AE signals for BN and GN in different PTs using the Breslow-Day test. GN and BN totally detected 33 positive AE signals. Among the positive signals we detected, 12 PTs were recorded in the drug label, and 21 were not. The result of the AE signal detected in the cardiac system (SOC) was shown in Figure 2, which contained seven PTs related to cardiac AEs recorded in the insert and two PTs not recorded. Among the evaluated nine PTs, three PTs, namely, cardiac arrest, ventricular tachycardia, and unstable angina, showed opposition between negative and positive signals. However, only angina unstable instability showed statistical significance (*p* < 0.001) according to the Breslow-Day test, suggesting a higher risk for GN. Furthermore, although both the BN and GN showed a positive ADR signal in atrial fibrillation and arrhythmia, the risk of the GN was significantly higher (*p* < 0.001).

**FIGURE 3:**
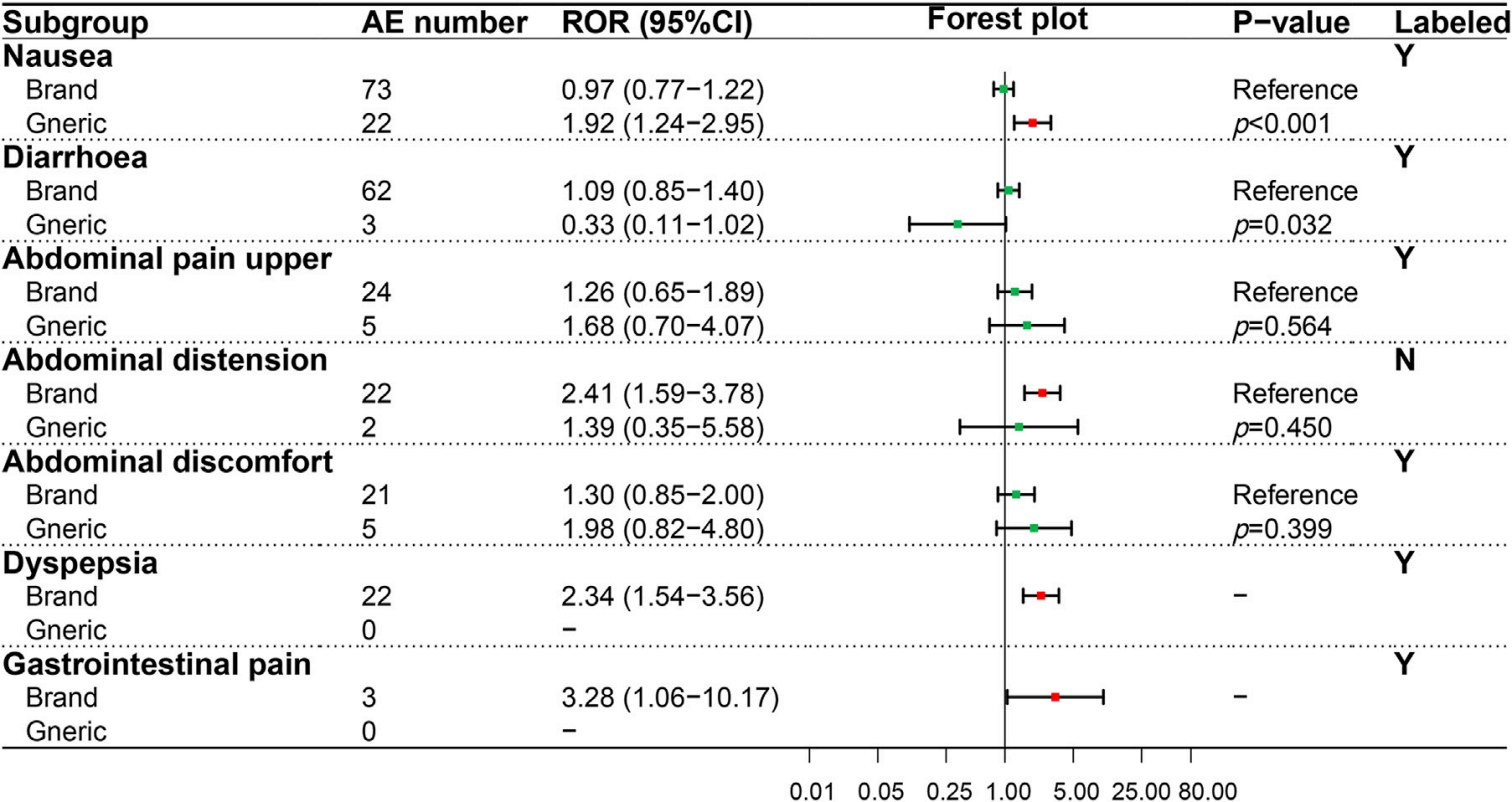
Comparison of detected AE signals for brand and generic nebivolol in the gastrointestinal system. Abbreviate: AE, adverse event; ROR, reporting odd ratio; CI, confidence interval. Note: Red points indicate positive signals and green points are opposite; the *p*-value results from the Breslow-Day test; label information comes from the US Food and Drug Administration (FDA) official website.

A similar analysis was performed on the gastrointestinal system (SOC), and seven PTs were evaluated (Figure 3). Results showed that the BN and GN only exhibited a significant difference in nausea (*p* < 0.001), an AE recorded in the package insert. In addition, although two positive signals, namely, abdominal pain, and vomiting, were only detected in BN, the risk difference between BN and GN cannot be compared due to the missing ADR signal in GN.

**FIGURE 4:**
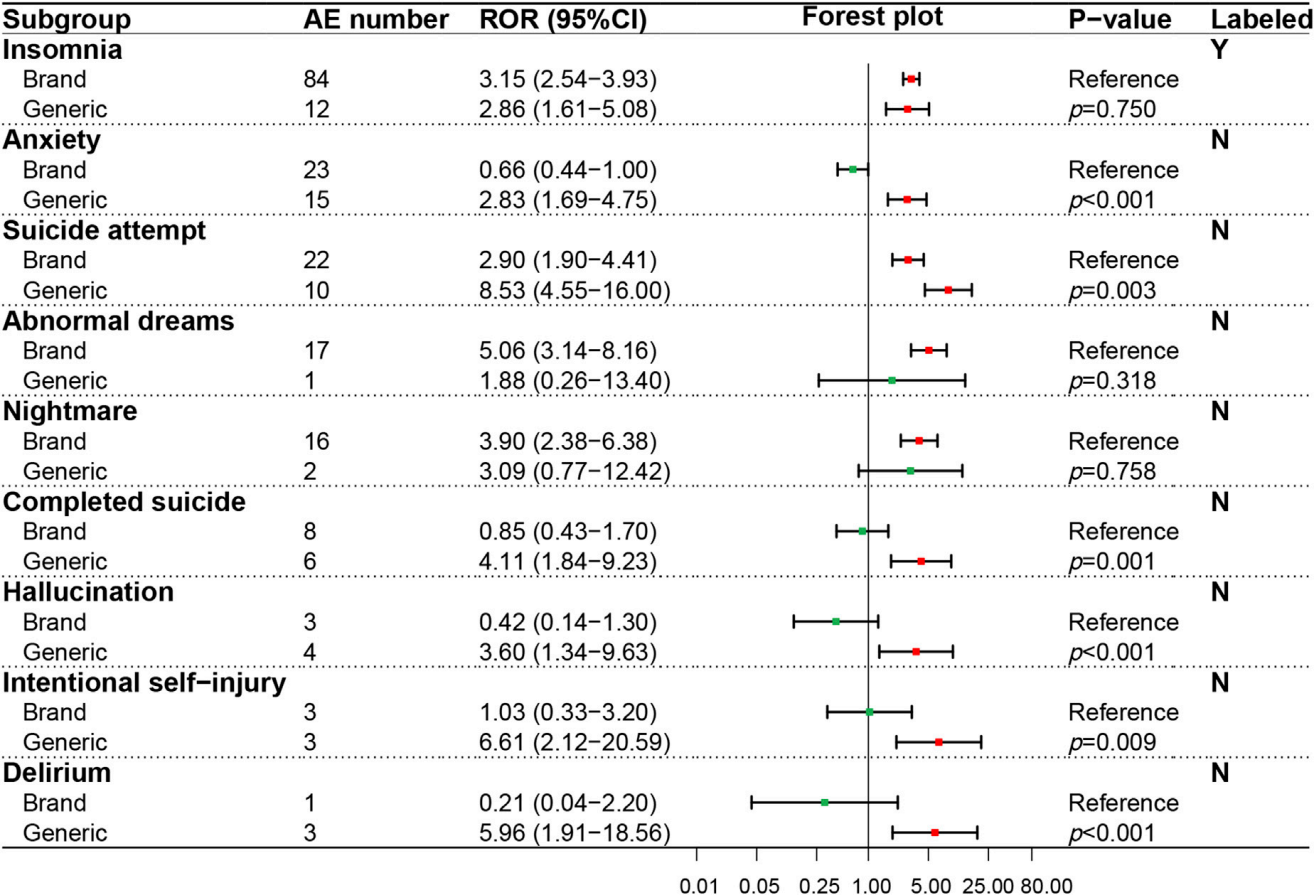
Comparison of detected AE signals for brand and generic nebivolol in the psychiatric system. Abbreviate: AE, adverse event; ROR, reporting odd ratio; CI, confidence interval. Note: Red points indicate positive signals and green points are opposite; the *p*-value results from the Breslow-Day test; label information comes from the US Food and Drug Administration (FDA) official website.

**FIGURE 5:**
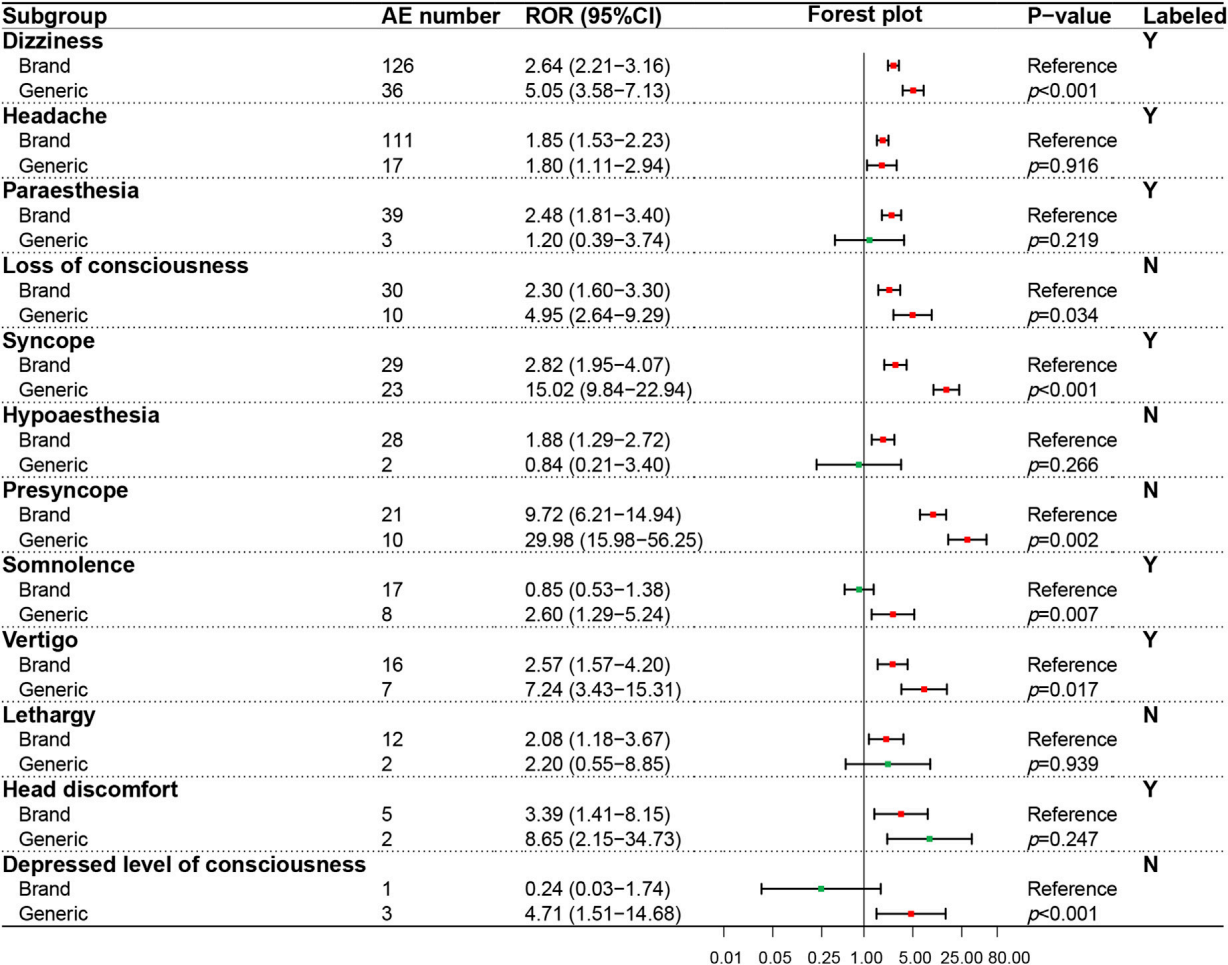
Comparison of detected AE signals for brand and generic nebivolol in the nervous system. Abbreviate: AE, adverse event; ROR, reporting odd ratio; CI, confidence interval. Note: Red points indicate positive signals and green points are opposite; the *p*-value results from the Breslow-Day test; label information comes from the US Food and Drug Administration (FDA) official website.

In the psychiatric system (SOC), the ADR signals of nine PTs were evaluated (Figure 4), in which only one PT (insomnia) was recorded in the package insert. On the whole, GN showed a higher ADR risk in the psychiatric system than BN, especially significant in anxiety (*p* < 0.001), suicide attempt (*p* = 0.003), completed suicide (*p* = 0.001), hallucinations (*p* < 0.001), intentional self−injury (*p* = 0.009) and delirium (*p* < 0.001). The ADR signal results detected of 12 PTs in the neural system (SOC) were shown in Figure 5, in which five PTs were not recorded in the package insert and showed potential risk. Similar to the psychiatric system (SOC), the overall ADR risk of GN in the neural system is higher than BN and showed significant differences in dizziness (*p* < 0.001), syncope (*p* < 0.001), presyncope (*p* = 0.002), somnolence (*p* = 0.007) and decreased level of consciousness (*p* < 0.001).

## 4 Discussion

Generic drugs constitute a sizeable portion of the marketplace, and a long-term safety evaluation of post-marketing generic drugs cannot be ignored (White, 2020). In this study, we reviewed AE reports associated with nebivolol in the FAERS database to obtain ADR risk information of GN and BN. Meanwhile, to explore the safety profile for the GN and BN, we conducted a disproportional analysis of four SOCs. In addition, we performed the heterogeneity tests for ADR signals using the Breslow-Day test. Our results showed that some potential ADRs of nebivolol were not recorded in the package insert, and there was a difference in the safety profile between BN and GN.

PT is a detailed description of the specific clinical manifestations, site of occurrence, and disease subtype of a disease or AEs, and it is also the recommended term level for analysis of pharmacovigilance data (Brown, 2004; Bousquet et al., 2014). For the positive ADR signal recorded in the drug label, such as bradycardia (brands, ROR = 23.37, 95% CI:19.42–28.12; generics, ROR = 31.73, 95% CI: 21.12–47.67), headache (brands, ROR = 1.85, 95% CI: 1.53–2.23; generics, ROR = 1.80, 95% CI: 1.11–2.94), our results were a kind of re-verification for these ADRs of nebivolol in pharmacovigilance perspective. Moreover, for the positive AE signals not recorded in the drug label, our result showed that some potential AE risks during nebivolol use might have been previously overlooked, which deserved further attention. For example, we detected an AE signal of unstable angina (PT) in cardiac disorders, which was also described in the published literature (Akkus et al., 2012). Unstable angina requires early intervention, and its common clinical symptom is chest pain, so health professionals should pay attention to the differential diagnosis of patients with emerging chest pain on nebivolol (Kalra et al., 2008; Akkus et al., 2012). In addition, we should pay attention to the psychiatric and neurological AE risk of nebivolol. Nebivolol is a highly fat-soluble drug that is relatively easy to cross the blood-brain barrier, and its physical and chemical properties determine that it has the potential effect on neurological and psychiatric systems (Kumar et al., 2007; Cruickshank, 2010). This study detected eight unrecorded positive AE signals in the psychiatric system (SOC) and five in the neurological system (SOC), respectively. In this regard, nebivolol’s high neurological and psychiatric AE risk can be partly attributed to its high lipid solubility, although further verification is needed.

In addition, to compare the AE risk difference between BN and GN, we performed the heterogeneity tests for AE signals using the Breslow-Day test. Our result showed that 15 GN-BN pairs have significant differences, which suggested that these AEs might be related to whether they are generics or brands. In these pairs, the ROR values of GN were all greater than those of BN, indicating that generic drugs were more likely to have these AEs. Although generic medications were theoretically equivalent to the originators, their actual performance in the clinical setting might not be as good as theoretical expectations (Bialer and Midha, 2010; Serebruany et al., 2019). Such a difference is explainable. On the one hand, original drugs are supported by much scientific research and safety data, while generic drugs are generally marketed based on pharmacological and bioequivalence only, lacking long-term safety and research in large samples (Meredith, 2003). On the other hand, the prescriptions of originator drugs are usually confidential, which leads to differences in the selection and dosage of excipients and the preparation process of generic drugs. As we know, excipients are chemical substances other than the active pharmaceutical ingredient (API), and they are added intentionally during the preparation of drugs to serve a specific purpose in the finished product, which have no effective pharmacological activity or impact therapeutic efficacy or safety ideally (Kalász and Antal, 2006). A study based on vitro experiments showed that the usage of excipients such as microcrystalline cellulose and starch could affect the properties of nebivolol tablets (Shaikh et al., 2010). Excipients might influence the release and (or) absorption of the API. If the increased release and (or) absorption of the API occurred in the clinical use of nebivolol, the patient may suffer from a higher risk of neurological and psychiatric disorders (Olawi et al., 2019). In addition, some excipients might even cause unexpected adverse reactions (Pifferi and Restani, 2003; Kalász and Antal, 2006; Rayavarapu et al., 2015). Therefore, it is necessary to continuously pay attention to the post-marketing safety of generic drugs and find out the related drug risks in time.

To our knowledge, our research is the first study focusing on the safety investigation of GN and BN from the pharmacovigilance perspective, which provides additional information on the safety of nebivolol in a large sample of the population and also provides data support for clinical medication decision-making. Meanwhile, our study also provides a low-cost, reliable, and convenient strategy to compare the safety profile difference between BN and GN. However, our study has some unavoidable limitations due to the inherent nature of pharmacovigilance database. Firstly, the inconsistency in time-to-market for BN and GN may have an unknown effect on the study results. For example, brands may detect more new signals due to marketed earlier, and generics with a shorter time-to-market may be affected by Weber’s effect resulting in higher values for some AE signals (Hoffman et al., 2014; Rahman et al., 2017a). Secondly, patient age, sex, comorbidities, drug dosage, and previous medical history may potentially influence the occurrence of AE. However, there is currently no well-established method that can be used to eliminate the influence of these factors on our results. Thirdly, the FAERS database runs on the basis of voluntary reporting, so underreporting, omissions, duplicate reporting, notoriety bias, and other situations may affect our results (Alatawi and Hansen, 2017; Neha et al., 2021). Fourthly, when we calculate the signal values for the BN or GN, the nebivolol data for the remaining group are grouped together to “other drugs”, which may have a potential influence in the result. Fifthly, our study is conducted based on the disproportional analysis, which can only indicate a statistical association between the drug of interest and AE of interest rather than a genuine causal relationship. Finally, there are no pharmacokinetic studies or clinical studies currently to support that BN is safer than GN, so the results in our study should be interpreted cautiously and further validation is needed.

## 5 Conclusion

Based on the review of safety data in the FAERS database, our study conducted a disproportional analysis for GN and BN in cardiac, gastrointestinal, neurological, and psychiatric systems. Our study suggested that certain potential AE might be more likely to occur with GN rather than BN, which provides extra information for the selection and clinical use of GN and BN in the real world and may contribute to ADR monitoring of nebivolol. However, it is particularly noteworthy that the detected AE signals only represent the statistical relationship for drug-AE combination, and the actual causal relationship requires further validation.

## Data Availability

The original contributions presented in the study are included in the article/Supplementary material, further inquiries can be directed to the corresponding authors.
